# Supplementary material for: Intracellular nucleosomes constrain a DNA linking number difference of −1.26 that reconciles the Lk paradox
Source: Nat Commun. 2018 Sep 28;9:3989. doi: 10.1038/s41467-018-06547-w (PMC6162219; doi:10.1038/s41467-018-06547-w)
Supplement: Supplementary file 1 — Supplementary Information [file 41467_2018_6547_MOESM1_ESM.pdf]

Supplementary Information

Intracellular nucleosomes constrain a DNA linking number difference of -1.26 that reconciles the *Lk* paradox

Segura et al.

A

Oligonucleotides to amplify genomic coordinates 461739 to 462736 (997 bp) that include TRP-ARS1

```
F:        5'GATAGAATTCGGTCGAAAAAAGAAAAGGAGAGGGCCAAGAGGGAG 3'
R:        5'AAGGCGCGCCAGCGGCCGCCATTCTCTTCAACAAGTTTGATTCC 3'
```

Oligonucleotides to amplify genomic coordinates 238194 to 238437 (243 bp) that include CEN2

```
F:     5' TATAGGATCCCATACATGAAGAATATAGGATCTGTATATCG  3'
R:     5' CATAGGTACCTAATAATAAATTAATCTTGAGCAAATTGGTCC 3'
```

B

YCp1.3 (1341 bp)

```
GCGGCCGCTGGCGCGCCGGATCCCATACATGAAGAATATAGGATCTGTATATCGTCGTCACCGACGTAAGGTTTTTTTGT
CAATTCTCGGTGAACAATTTTTTGCAAGAAATATATTGATACTTCTTGTTGAAATATTTTTCTTTTTCGGAAAACAAAAA
CATAAAATAATTATTTAAGAAAAAAAATTAATTAATAAATTTAAATAGAAAATCTTTTTTACTTAATAATAATTAAATAA
ATAAGTCACATGATTTATGTAGGACCAATTTGCTCAAGATTAATTTATTATTAGGTACCAAGATAATAGTAACCCGGGTT
AATAAGTGTATTTGAGATAGAATTCGGTCGAAAAAAGAAAAGGAGAGGGCCAAGAGGGAGGGCATTGGTGACTATTGAGC
ACGTGAGTATACGTGATTAAGCACACAAAGGCAGCTTGGAGTATGTCTGTTATTAATTTCACAGGTAGTTCTGGTCCATT
GGTGAAAGTTGCGGCTTGCAGAGCACAGAGGCCGCAGAATGTGCTCTAGATTCCGATGCTGACTTGCTGGGTATTATATG
TGTGCCCAATAGAAAGAGAACAATTGACCCGGTTATTGCAAGGAAAATTTCAAAGTCTTGTAAAAGCATATAAAAATAGT
TCAGGCACTCCGAAATACTTGGTTGGCGTGTTTCGTAATCAACCTAAGGAGGATGTTTTGGCTCTGGTCAATGATTACGG
CATTGATATCGTCCAACTGCATGGAGATGAGTCGTGGCAAGAATACCAAGAGTTCCTCGGTTTGCCAGTTATTAAAAGAC
TCGTATTTCCAAAAGACTGCAACATACTACTCAGTGCAGCTTCACAGAAACCTCATTCGTTTATTCCCTTGTTTGATTCA
GAAGCAGGTGGGACAGGTGAACTTTTGGATTGGAACTCGATTTCTGACTGGGTTGGAAGGCAAGAGAGCCCCGAAAGCTT
ACATTTTATGTTAGCTGGTGGACTGACGCCAGAAAATGTTGGTGATGCGCTTAGATTAAATGGCGTTATTGGTGTTGATG
TAAGCGGAGGTGTGGAGACAAATGGTGTAAAAGACTCTAACAAAATAGCAAATTTCGTCAAAAATGCTAAGAAATAGGTT
ATTACTGAGTAGTATTTATTTAAGTATTGTTTGTGCACTTGCCTGCAGGCCTTTTGAAAAGCAAGCATAAAAGATCTAAA
CATAAAATCTGTAAAATAACAAGATGTAAAGATAATGCTAAATCATTTGGCTTTTTGATTGATTGTACAGGAAAATATAC
ATCGCAGGGGGTTGACTTTTACCATTTCACCGCGATGGAATCAAACTTGTtGAAGAGAATG
```

GCGGCCGC – NotI
GGCGCGCC – AscI
GGATCC – BamHI
GGTACC – KpnI
GAATTC - EcoRI
AAGCTT – HindIII
CEN2 elements: CDEIII CDEII CDEI
TRP1-ARS1 genomic fragment
TRP1 coding sequence - ARS1 sequence

Supplementary Fig 1. Construction of YCp1.3. **A**. PCR oligonucleotides used to amplify the TRP-ARS1 segment and the CEN2 of *S.cerevisiae*. **B.** Complete base pair sequence (5' to 3') of the YCp1.3 circle. Relevant restriction sites and the functional elements of YCp1.3 are color coded as indicated.

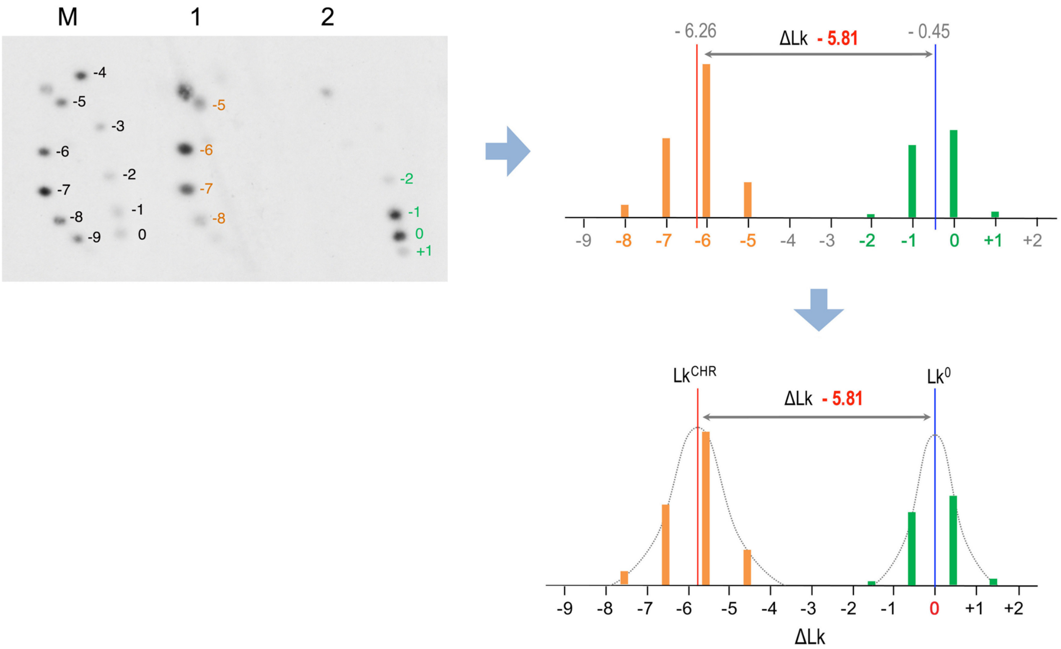

Supplementary Fig 2. Analysis of *Lk* distributions and calculation of Δ*Lk*. Top-left: *Lk* markers (lane M) helped to count unambiguously the number of topoisomers between *Lk* distributions (lanes 1 and 2) resolved in the same 2D gel. An integer Δ*Lk* value (colored) was initially assigned to each *Lk* topoisomer of the minichromosome (lane 1) and the relaxed DNA (lane 2). To this end, the most intense topoisomer of relaxed DNA was given the value Δ*Lk*=0. Top-right: From the individual topoisomer intensities and assigned Δ*Lk* values, the mean of each *Lk* distribution was calculated (grey). The Δ*Lk* between the *Lk* distributions was then obtained by subtracting their means. Bottom: These results were illustrated in the figures by plotting the intensity of individual *Lk* topoisomers of minichromosomes and relaxed DNA along a scale of Δ*Lk* units (x-axis), in which the value Δ*Lk*=0 was corrected to the mean of the Lk distribution of the relaxed DNA (Lk^0). The mean of the *Lk* distribution of the minichromosome (Lk^{CHR}) was thereby Δ*Lk*. This presentation of the data facilitated the comparison of distinct minichromosome clones, since all were aligned by Lk^0.

A

Adaptor with BamH1 cohesive-end ligatable but not recutable

```
5′      *GGACGATTACAGCTACGTG       3′
3′      TCCTGCTAATGTCGATGCACCTAG   5′
```

Adaptor with Asci cohesive-end ligatable but not recutable

```
5′      *CGTTCGGATCCGTTTAAACGTGAAGAGGTAACTCAT      3′
3′      TGCAAGCCTAGGCAAATTTGCACTTCTCCATTGAGTAGCGC  5′
```

B

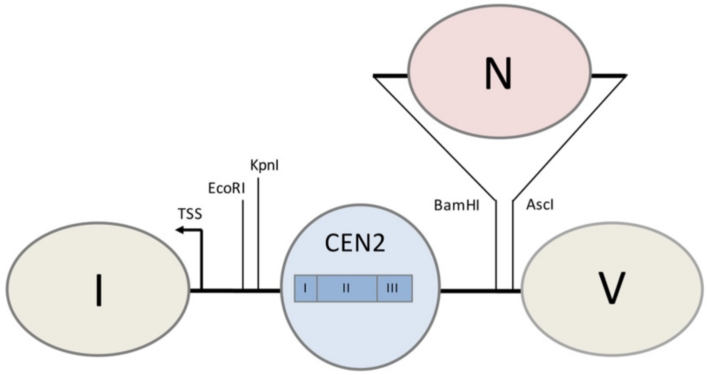

Supplementary Fig 3. Inclusion of a mononucleosomal library into YCp1.3. A. Adaptors ligated to A-tailed mononucleosomal DNA fragments. Note that the BamH1 and Asc1 cohesive ends could be ligated but not recutted. This strategy facilitated their insertion in between the BamH1 and Asc1 sites of YCp1.3. **B.** Position of the nucleosome library with respect to nucleosome V and the CEN2 elements of YCp1.3.

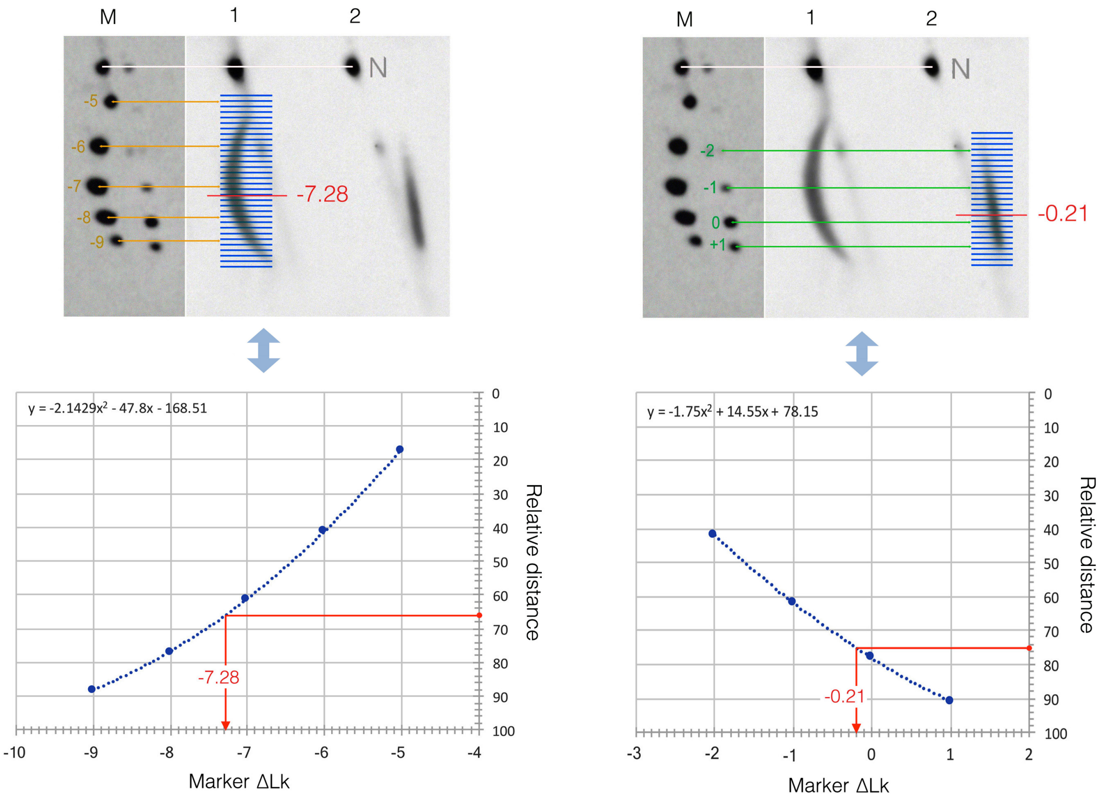

Supplementary Fig 4. Analysis of the pooled *Lk* distributions and calculation of Δ*Lk*. An integer Δ*Lk* value was assigned to individual *Lk* topoisomers of the marker (lane M, overexposed) as explained in Supplementary Figure 2. The signals of pooled *Lk* distributions of the minichromosomes (top left, lane 1) and of their relaxed DNAs (top right, lane 2) were quantified by bins (blue sections) and their mean was determined (red marks). In the bottom graphs, the gel position of the means (red dots) was interpolated with that of the *Lk* topoisomers of the marker (blue dots) to obtain the Δ*Lk* value of the means in the marker frame. Y-axes are relative migration distances to the nicked DNA circles (N) in the first gel dimension. X-axes are the integer Δ*Lk* values assigned to the marker. The Δ*Lk* between the pool of minichromosomes and their relaxed DNAs was determined by subtracting the Δ*Lk* values so obtained in the marker frame (Δ*Lk* = -7.28 -(-0.21) = -7.07).

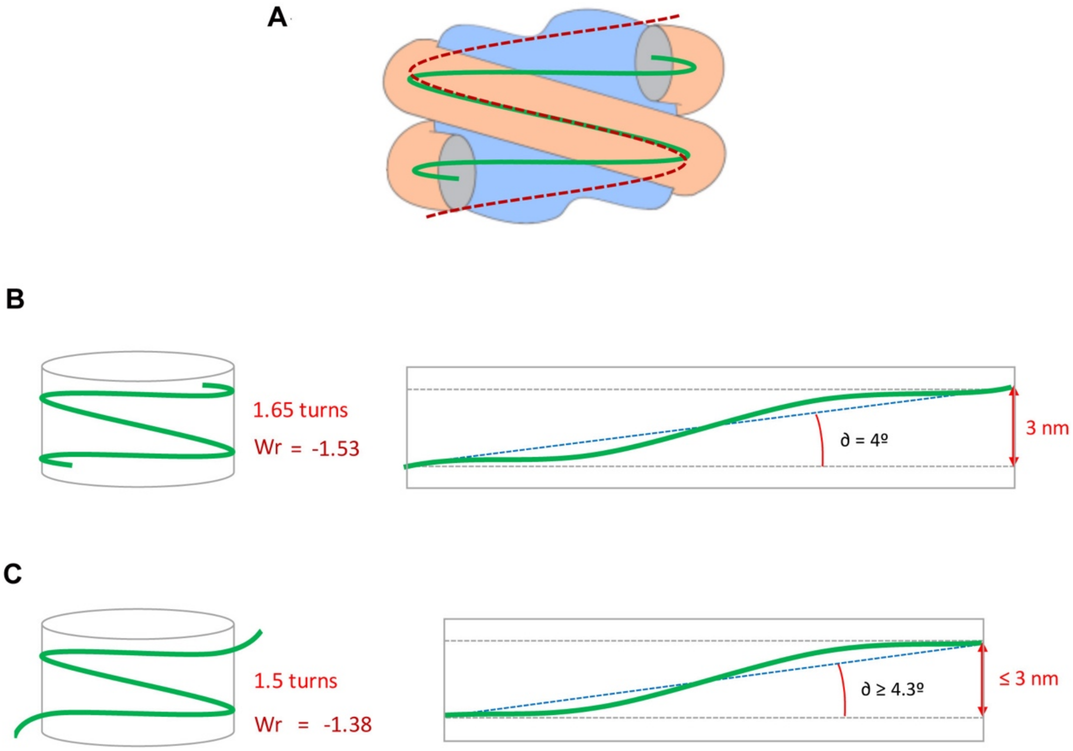

Supplementary Fig 5. Average pitch angle and effective writhe of nucleosomal DNA. **A**. The turning of DNA around the histone core (green) does not follow a regular helical path (dashed red). The average pitch angle (∂) is then determined from the helical height achieved by the entire contour of wrapped DNA. The writhe is then given by $Wr = n\,(1 - \sin \partial)$, where n is the number of helical turns. **B.** If n≈1.65 (as in crystallized nucleosomes and chromatosomes), *Wr* = -1.53. **C.** If n = 1.5 (due to partial unwrapping or breathing motions of the core DNA), *Wr* = -1.38.
