# Supplementary material for: Intracellular nucleosomes constrain a DNA linking number difference of −1.26 that reconciles the Lk paradox
Source: Nat Commun. 2018 Sep 28;9:3989. doi: 10.1038/s41467-018-06547-w (PMC6162219; doi:10.1038/s41467-018-06547-w)
Supplement: Supplementary file 3 — Description of Additional Supplementary Files [file 41467_2018_6547_MOESM3_ESM.pdf]

Description of Additional Supplementary Files

File Name: Supplementary Data 1
Description: Genomic coordinates of the mononucleosome library
